# Vaccinating for Whom? Distinguishing between Self-Protective, Paternalistic, Altruistic and Indirect Vaccination

**DOI:** 10.1093/phe/phaa005

**Published:** 2020-03-11

**Authors:** Steven R Kraaijeveld

**Affiliations:** Wageningen University & Research

## Abstract

Preventive vaccination can protect not just vaccinated individuals, but also others, which is often a central point in discussions about vaccination. To date, there has been no systematic study of self- and other-directed motives behind vaccination. This article has two major goals: first, to examine and distinguish between self- and other-directed motives behind vaccination, especially with regard to vaccinating for the sake of third parties, and second, to explore some ways in which this approach can help to clarify and guide vaccination debates and policy. I propose conceiving of vaccination in terms of three basic elements: the vaccination decision-maker, the vaccine recipient and the primary beneficiary. I develop a taxonomy based on the relations between these elements to distinguish four kinds of vaccination: self-protective, paternalistic, altruistic and indirect. I finally discuss the case of human papillomavirus vaccine regulation for men and women to show how each kind of vaccination is associated with and raises specific ethical questions.

## Introduction

Preventive vaccines represent one of the most significant advances in public health over the past 100 years (Feemster, 2018). Recent quantitative analyses have estimated that mass vaccination programs prevented over 100 million cases of contagious diseases since 1924 in the USA (Van Panhuis *et al.*, 2013) and averted around 9000 deaths of children born between 1903 and 1992 in the Netherlands (Van Wijhe *et al.*, 2016). Despite the overwhelming overall success of vaccination, parents in the industrialized world increasingly refuse routine childhood vaccines (Omer *et al.*, 2009; Navin, 2016). Vaccination thus remains a contested but vital issue for individual and public health; so important, in fact, that the years 2011–2020 were named "decade of vaccines" by the World Health Organization (2013).

Importantly, vaccination can protect not merely the vaccinated individual, but also others. It can do so directly, by preventing transmission to others (Verweij, 2005; Orenstein and Ahmed, 2017), indirectly, by contributing to herd immunity (Metcalf *et al.*, 2015), or through a combination of direct and indirect effects. Ethical reflection on vaccination has often centered on the question of whether or not (certain types of) vaccines ought to be mandatory (Galanakis *et al.*, 2013; Dubov and Phung, 2015; Pierik, 2018) and on whether particular vaccination programs are justified (Krantz *et al.*, 2004; Houweling *et al.*, 2010; Schwartz and Caplan, 2011). In discussions about the protective effects of vaccination, self-interest and the interests of others are usually highlighted. The rationale behind encouraging or regulating vaccination, for instance, is often the protection of third parties (Giubilini, 2019). To date, however, there has been no systematic study of the self- and other-directed motives behind vaccination. Yet vaccination debates are often implicitly if not explicitly about dynamics between the self and others within the context of individual vaccination decisions and vaccination programs.^1^ It is important to distinguish between self- and other-directed effects of vaccination, in order to clarify and address questions about, for example, the extent to which people ought to be altruistic in their vaccination behavior, or the degree to which governments should regulate vaccination in order to protect third parties. The relations between self- and other-directed motives behind vaccination have potential ethical implications for individual vaccination decisions as well as for public health policy. The possibility of vaccinating for the sake of others presents itself when vaccines are able to protect people other than those who are vaccinated. It is this phenomenon especially that I wish to explore. In order to examine vaccination that is directed toward benefiting others, it is useful first to differentiate it from vaccination that more fundamentally concerns the self.

I assume that the overarching motive of vaccination as such is protection against (a specific) disease.^2^ In order to recognize the more specific self- and other-directed motives behind vaccination, I conceive of vaccination as emerging from a combination of (i) the vaccination decision-maker, (ii) the vaccine recipient and (iii) the primary beneficiary of vaccination.^3^ Inquiry into ethical questions surrounding self- and other-directed motives behind vaccination will benefit from a better understanding of the relations among these three elements.

Taking up this task, I develop a taxonomy of four kinds of vaccination that I call self-protective vaccination, paternalistic vaccination, altruistic vaccination and indirect vaccination.^4^ These kinds of vaccination are descriptive, in that they map onto existing vaccination practices (as I will illustrate). At the same time, they may function as ideal types, and can explicitly be adopted as vaccination policies, for instance by governments seeking to regulate vaccination for the sake of third parties.

While the motive of vaccination generally is protection against disease, for each of the four kinds of vaccination the specific motive is to realize the benefits of vaccination for a particular person or group (i.e., the primary beneficiary). While the general motive is thus the same for each kind of vaccination, how that motive is ultimately realized—that is, by, through and for whom—will, as I hope to show, determine much of the ethical nature of each kind of vaccination. For example, while protecting third parties may be achieved through either altruistic or indirect vaccination, these approaches are not equivalent in the ethical concerns that they raise.

I discuss the concrete case of human papillomavirus (HPV) vaccine regulation for men and women in order to illustrate the value of the proposed taxonomy and to explore how applying the different kinds of vaccination can help to advance vaccination debates.

## Self-Protective Vaccination

The first kind of vaccination that I distinguish involves an individual agent, autonomous in her ability to make decisions, who is motivated to protect herself against becoming ill. With self-protective vaccination, the agent, aware that there are certain diseases that she may contract, and conscious of the fact that there are vaccinations to be had that will probabilistically protect her against those diseases, decides to vaccinate.^5^

The motive for this kind of vaccination is self-protection, since the primary benefits of vaccination (i.e., protection against disease) are sought for oneself. The person who decides to be vaccinated, then, is identical to the person who ultimately receives the vaccine, and the primary benefits and burdens associated with vaccination are accordingly borne by the one who makes the decision. If the perceived benefits of vaccination outweigh the perceived burdens to the agent, it may be assumed that they will go ahead and vaccinate.

An example of self-protective vaccination is an adult who decides to get a tetanus shot after cutting her hand on a rusty nail. The tetanus booster is explicitly sought out to prevent serious illness. More generally put, this category includes any case where someone is vaccinated of their own volition in order to protect their own person, and where the health of others is not directly at stake.

Compared with other kinds of vaccination, the ethics of self-protective vaccination is relatively constrained, since questions concerning moral duties, obligations and so on occur most pressingly at an interpersonal level, where the interests of one or more persons meet and potentially conflict with those of others. Unlike other kinds that I will discuss later, questions about respecting autonomy are unlikely to arise for self-protective vaccination, since deciding to vaccinate to protect oneself presumably requires an ability to make autonomous decisions. It seems implausible, for example, that an infant will seek a tetanus shot on her own initiative. There are nevertheless at least two areas in which ethical questions about self-protective vaccination arise.

First, governments may have an obligation to protect the basic conditions for public health (Verweij and Houweling, 2014), which includes ensuring equitable access to vaccination. Given various conditions of need, safety and scarcity of resources, the particular selection of vaccines that are included in collective vaccination programs—including vaccines that only seem to offer self-protection—is a matter for ethical debate.

Second, human beings may have duties to prevent harm to themselves (negatively stated), or to promote their own health and well-being (positively stated). These duties may be understood within the domain of prudence, which seems generally to be served by self-protective vaccination. Prudential arguments about whether and how comprehensively one ought to vaccinate in order to protect oneself against diseases will therefore be foregrounded. One might vaccinate in order to be healthy for others (e.g., if one has a duty of care). However, when the primary motive is to benefit others (even if through self-care), then, for reasons that will become clear later, this is best understood not as self-protective but as altruistic vaccination.

That being said, for questions about what human beings might owe others, we have to look to different kinds of vaccination—that is, to instances of vaccination whose decisional and motivational structures involve other agents.

## Paternalistic Vaccination

The second kind of vaccination fundamentally implicates more than one individual person. Paternalistic vaccination occurs when an autonomous agent, aware that there are diseases that another person may contract, and conscious of the availability of vaccines that will protect that person against the deleterious effects of those diseases, makes a decision for the latter to be vaccinated.^6^

The motive for this kind of vaccination is the direct protection of others for their own sake and circumscribed to those others. One must be careful here not to confuse paternalistic vaccination with indirect vaccination, which I will describe later. Without getting ahead, what must be noted here is that paternalistic vaccination, unlike indirect vaccination, takes as its primary motive protection against disease for the person who is vaccinated. Of course, motives may overlap, especially when the effects of a vaccine will protect the vaccinated person and third parties to relatively similar degrees. There is bound to be some degree of overlap between categories; for instance, altruistic vaccination will usually also protect the vaccinated person (thus potentially including a self-protective motive). What matters for my account, however, is the primary motive for the vaccination decision. In any case, as Broadbent (2019: 37) puts it, ‘[l]ack of clarity at the border does not obviate the value of the distinction’.

An important further consideration for paternalistic vaccination is whether or not the person for whom the vaccination decision is made is autonomous.^7^ An example of paternalistic vaccination for not or not fully autonomous persons is when a parent ensures that their child receives her tetanus shots. An example of paternalistic vaccination for autonomous persons, on the other hand, is when this same decision is taken and enforced by governments for citizens of legal age. The latter case negatively affects autonomy, since the vaccination decision is taken out of the hands of persons who are otherwise autonomous in their capacity to make vaccination decisions. When governments make the decision for citizens to vaccinate, thereby endorsing a form of paternalistic vaccination, the ‘decision’ may take various forms and may range from less to more coercive—from milder and relatively more autonomy-respecting measures like persuasion and nudging, to incentives, fines and more drastic measures like compulsion (cf. Giubilini, 2019). The more coercive the measure, the less freedom there is for a citizen to take her own decision. This is where paternalistic vaccination is distinguished from self-protective vaccination: in the former kind, the decision is imposed on the vaccinating person (e.g., through government regulation), while in the latter kind, the decision is freely taken by the vaccinating person.

More generally stated, paternalistic vaccination encompasses any case in which an agent decides for another person to be vaccinated, to the explicit benefit of the latter and with no one else’s well-being substantially at stake. The agent who makes the decision to vaccinate is, therefore, distinct from the person who is vaccinated, so that the main vaccination benefits and burdens are accordingly borne not by the agent from whom the decision stems, but by the person who receives the vaccine. Although there may be incidental benefits and burdens for the decision-making agent, these are not decisive.^8^

Important ethical considerations associated with paternalistic vaccination are likely to cluster around the responsibility and authority of the agent who makes the vaccination decision and are primarily steered by whether or not the person whom the vaccination decision affects is autonomous. This is not to suggest that overruling autonomy is intrinsically ethically unacceptable. There might be good reasons for paternalism when it comes to behavior that negatively affects health (e.g., Conly, 2013a,b), which may also apply to paternalistic vaccination, so that autonomy may sometimes justifiably be overridden. When potential subjects of paternalistic vaccination are autonomous, this simply means that questions about whether paternalism is justified will be most pertinent and that the requirements for justification itself will be more stringent compared with cases where subjects are not autonomous. Furthermore, it must be noted that paternalism is unlikely to be an all-or-nothing approach. Autonomy may be reduced in various ways and to different degrees, which also holds for paternalism in the area of vaccination policy.

When subjects are not autonomous, as in the case of children vaccinated by their parents, relevant ethical concerns are those that apply whenever decisions are made on behalf of someone who is not or not fully autonomous, when the latter’s health and well-being are substantially at stake. The principles of nonmaleficence and beneficence (Beauchamp, 2003; Beauchamp and Childress, 2012) are likely to take center stage here, as guides and measures to decisions about what is best for those who cannot make fully informed decision for themselves.

Since the crux of paternalistic vaccination is that a vaccination decision is made for another person—for that person’s benefit—the agent making the decision assumes a critical role, because the moral weight of the decision and its consequences ultimately rests on them.

## Altruistic Vaccination

Altruistic vaccination involves an autonomous agent who, conscious of the fact that there are diseases that others may contract, and aware that there are vaccinations to be had that would protect others from the noxious effects of those diseases, decides to be vaccinated.

The primary motive for this kind of vaccination is thus to protect other people against disease through one’s act of vaccination. In vaccination of this kind, the agent who makes the decision to vaccinate is identical to the agent who receives the vaccine (as in self-protective vaccination). However, the primary benefits of vaccination are directed toward someone other than the agent being vaccinated (unlike in self-protective vaccination).

The concept of altruism has been used in myriad ways across a number of disciplines (Scott and Seglow, 2007). At its core it is an act or desire to benefit someone other than oneself for the other person’s sake (Kraut, 2009). I adopt a moderate view of altruism, positioned between the strong view in which an act is only altruistic when it comes at a net cost to the actor, and the weak view in which it is sufficient that an act be motivated, at least in some part, by the fact that it benefits others (Kraut, 2016). Altruism does not necessarily entail self-sacrifice (although it might). It is enough that the desire to benefit others underlies one’s act of vaccination as a primary motive for it to constitute a case of altruistic vaccination. After all, vaccination can and often does confer benefits on the vaccinated individual as well as on those whom they seek to protect (and even on people not explicitly considered in the vaccination decision, for instance through herd immunity effects). On my account, it is sufficient that other-directed considerations primarily motivate an act of vaccination for it to be a case of altruistic vaccination.

As in the case of self-protective vaccination, concerns about respecting autonomy are unlikely to play an important role in altruistic vaccination, since the latter presupposes an ability to make autonomous decisions to decide to vaccinate for others. It is unlikely, for example, that an infant will seek a flu shot on her own account in order to protect others. There may be a gray area where children not entirely autonomous in their decision-making could decide to vaccinate for others. I leave this as an open question. However, when there is really no decision-making autonomy to speak of (e.g., in infants or severely mentally disabled people), then it seems to me that would-be altruistic vaccination will invariably be indirect vaccination (to be discussed in the following section). The decision will come from someone else.

As will become clear from the following examples, those who might benefit from altruistic vaccination can range from one particular, identifiable person to a more diffuse group of people.^9^

Through the practice of cocooning, people in close contact with newborns and infants—too young to be fully vaccinated—are immunized (Healy *et al.*, 2011; Urwyler and Heininger, 2014). This strategy is a case of altruistic vaccination when the decision is primarily intended to protect newborns and infants against becoming ill.

In the case of maternal immunization, levels of antibodies may be boosted during pregnancy so as to protect newborns from diseases that are caused by pathogens in the perinatal period, at least until the infant is old enough to be vaccinated (Munoz, 2018). When maternal immunization is chiefly directed toward protecting the health of the future child, this is an example of altruistic vaccination.^10^

Healthcare workers (HCW) may be immunized to protect high-risk groups of patients (e.g., the severely immuno-compromised) with whom they enter into close contact (Galanakis *et al.*, 2013). There may be good arguments for mandatory vaccination of HCW against influenza (Van Delden *et al.*, 2008), but we should consider it altruistic vaccination when HCW freely decide to vaccinate for the sake of patients.

Having offered examples of altruistic vaccination, what follows is a discussion of ethical considerations that these and other cases prompt. When one seeks to benefit others through vaccination, this constitutes a form of altruistic behavior that brings to light different moral concepts and ethical questions from those associated with, for instance, self-protective or paternalistic vaccination.

Altruism and freedom are intimately related, in that altruism seems to require freedom because it depends on the right kind of self-chosen motive (Seglow, 2004). Once altruism is institutionalized, however, a tension emerges because the motive to engage in altruistic acts is no longer free; it becomes tied to compliance with external demands. My account of vaccination accommodates this tension as follows: when the decision to vaccinate stems from the person who is vaccinated, then this is properly understood as altruistic vaccination. If, on the other hand, the decision is imposed from outside, then, as will be discussed in the next section, this is better conceived as indirect vaccination. The mere fact that people sometimes do vaccinate for the well-being of others (e.g., Betsch, 2014) is of course insufficient by itself to ground a normative account. However, whether and to what extent people ought to vaccinate altruistically is a different matter; it is central to the ethics of altruistic vaccination.

One objection to this reasoning might be that it is not always clear-cut whether someone vaccinates out of purely altruistic motives (Verweij *et al.*, 2016). For instance, a person may be conforming to social norms or peer pressure, even without government mandates. All the same, while degrees of freedom may vary, as long as the decision ultimately resides with the person who is vaccinated, then this is a case of altruistic vaccination. The moment that vaccination for others is imposed on people—whether by governments, through social pressure, or by any other means—it is no longer altruistic, and should not be viewed or explained as such.

Altruistic vaccination is an important example of vaccination for the sake of others, but it does not cover the whole range of the concept. Given that having an altruistic motive is a precondition for altruism, decisions that do not derive from the right kind of motive cannot be accommodated by altruistic vaccination—even if there are benefits to others. Another kind of vaccination must be distinguished.

## Indirect Vaccination

The final kind of vaccination involves a decision-maker who is neither the person who is vaccinated nor the one who receives the primary benefits of vaccination. Specifically, indirect vaccination^11^ entails a decision-maker who, recognizing that there are diseases that (a member of) a group may contract, and, knowing that there are vaccinations to be obtained by nonmembers that would protect (a member of) that group from the harmful effects of those diseases, decides that a nonmember should be vaccinated.

The primary motive for this kind of vaccination is to protect an individual or a group of people against disease through others. Indirect vaccination, then, is like paternalistic vaccination by virtue of the separation between vaccination decision-maker and recipient, and like altruistic vaccination in that the primary benefits of vaccination are extended to someone other than the one being vaccinated. In this latter sense, it is a form of vaccination for the sake of others. Nevertheless, because the decision to vaccinate does not come from the person who is vaccinated, it cannot be considered altruistic; instead, it is a form of vaccination indirectly meant to benefit others.

The identity of the decision-maker may, at least at first glance, appear to be more obscure for indirect vaccination than for other kinds. This is partially due to the nature of indirect vaccination, which will usually be instantiated at the level of institutions—unlike other kinds of vaccination, which tend to involve clearly identifiable individuals. To clarify: in the context of indirect vaccination, I take a vaccination decision to mean, loosely, an explicit decision to try to achieve the aims of vaccination—protection against disease. Decisions may take place at various levels, from companies, organizations and institutions (e.g., hospitals requiring that HCW are vaccinated for the sake of patients) to governments (e.g., mandatory vaccination programs to protect vulnerable citizens). Any form of pressure may come into play, from punishment to reward. The methods used to enforce the vaccination decision will be part of the ethical evaluation of indirect vaccination, rather than intrinsic to it. It must be noted that indirect vaccination is not necessarily confined to the level of institutions. For instance, parents may decide to vaccinate one of their two children for the sake of the other child. The decision here derives from the parents—not the vaccinated child—and the primary benefits do not go to the vaccinated child.

When the decision to vaccinate for the sake of others is freely taken by an individual, we should speak of altruistic vaccination, so that a certain amount of persuasion may be compatible with an altruistic approach. However, when the decision takes place beyond the individual, and especially when enforcing it becomes a possibility and practice, we enter the terrain of indirect vaccination. The distinction may not always be clear-cut, and it may be contested—yet it is nevertheless important to draw.

An example may be useful here. So far, I have not mentioned the possibility of vaccination that aims at achieving or maintaining herd immunity, for instance for an infectious disease like influenza (Plans-Rubió, 2012). Herd immunity effects necessarily reach others beyond the vaccinated individual. Vaccination for herd immunity may therefore provide an instance of either altruistic or indirect vaccination, depending on the primary motive and where the decision ultimately lies. Under which category it will ultimately fall depends, I have argued, on whether people altruistically decide to vaccinate in order to contribute to herd immunity (altruistic vaccination), or whether this decision comes from elsewhere and is required in some way (indirect vaccination). While it is possible, in principle, that an individual vaccinates primarily for the sake of herd immunity, this is unlikely in practice to be a primary motive for individuals. Herd immunity is an abstract and rather elusive goal of vaccination, which limits its motivating force—although governments may, of course, attempt to strengthen its motivating force directly, for instance through public campaigns that appeal to the importance of herd immunity and of individuals altruistically vaccinating in order to help achieve it. Furthermore, an individual person’s act of vaccination is highly unlikely to make a difference to whether or not herd immunity is actually achieved (Giubilini, 2019). It is therefore improbable that sufficiently large herd immunity effects can be achieved by merely relying on peoples’ inclinations toward altruistic vaccination. Furthermore, there is the related issue of fairness in the distribution of the burdens of vaccination, which is likely to fall disproportionately on those individuals who vaccinate altruistically, and which may require that governments have to undertake an indirect vaccination approach as a matter of justice (Giubilini, 2019). At a socio-political level, therefore, relying on altruistically vaccinating individuals may be both impractical, given the aims of vaccination, and unfair, given that others would end up enjoying the benefits of herd immunity without bearing any of the burdens.

A further consideration for indirect vaccination is whether or not the person for whom the vaccination decision is made is autonomous. An example of indirect vaccination for persons who are not fully autonomous is when one group of children is required to be vaccinated against measles for the benefit of yet another group of children (e.g., those who are too young to be vaccinated). A case of indirect vaccination for autonomous persons is when professionals are required to vaccinate for the sake of people for or with whom they work. Practically speaking, questions about autonomy will play a part even if the direct subjects of indirect vaccination are not themselves autonomous, because those who are not capable of making autonomous vaccination decisions will usually be cared for by others who are authorized to make decisions on their behalf. In the case of young children, it will often be the autonomy of parents that is at stake. Governments could, for instance, require parents to vaccinate their children even if the primary motive is not to benefit their particular children. In such cases, parental autonomy seems to be at stake.

Indirect vaccination is the most complex and demanding kind of vaccination in terms of ethical justification. It has the highest threshold for acceptability, not only because it tends to override autonomy (Beauchamp and Childress, 2012), but also because it is generally more difficult to defend imposing burdens associated with vaccination, like side effects, inconvenience, money or time (Fine *et al.*, 2011), when the primary benefits do not go to those who bear them. Removing the decision to vaccinate from the realm of individual discretion may, however, provide a solution to low vaccination uptake and may serve to achieve or preserve herd immunity. These considerations will ultimately require ethical justification beyond that associated with the previously considered kinds of vaccination. For it is one thing to encourage someone to vaccinate for the sake of others, yet it is quite another thing to require them to do so through coercive measures. Where altruism is untenable for bringing about the benefits of vaccination, aside from the previously considered issue of fairness, a normative conception of solidarity (e.g., Carson and Flood, 2017) and a moral duty to contribute to public goods (e.g., Verweij and Houweling, 2014) are potential justifications for why governments could opt for indirect vaccination. As for the shape that indirect vaccination might take, the vaccination intervention ladder proposed by Giubilini (2019) offers a useful guide; interventions involving persuasion are compatible with altruistic vaccination, since they preserve freedom of choice, while financial incentives and disincentives already begin to erode altruistic vaccination and bring one within the realm of indirect vaccination.

Individuals, institutions or governments may wish to protect certain individuals or groups of people against disease; in order to do so, what must be considered first and foremost is to whom the benefits and burdens of vaccination are directed, especially and most pressingly when decision are made for others through an indirect vaccination approach.

## Case Study: HPV Vaccination

**Figure 1.**
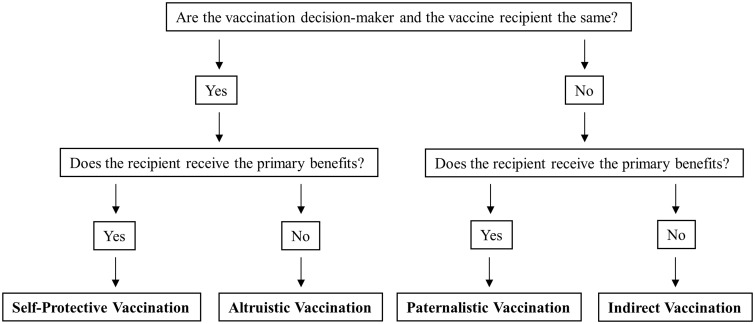
Taxonomy of four different kinds of vaccination.

Since most decisions about vaccination take place at the level of government policy, I want to focus the discussion on vaccine regulation. More specifically, I want to explore how my taxonomy contributes to current ethical debates by examining some tensions that arise when governments consider regulating vaccination for the sake of others, which, as I have tried to show, can be conceived of as altruistic (when the decision comes from the person vaccinating) or as indirect (when the decision comes from elsewhere). See Figure 1 for an overview of the four different kinds of vaccination.

Let us take the case of a government considering not only vaccinating girls against HPV, but also boys. HPV is among the most common sexually transmitted infections; while most infections are temporary and remain subclinical, persistent infection can lead to cancers—including cervical cancer, which for cancer incidence and mortality in women ranks fourth worldwide (Bray *et al.*, 2018). Evidence of the safety and effectiveness of HPV vaccines currently on the market is highly robust (Schiller *et al.*, 2012; Donken *et al.*, 2018; Sipp *et al.*, 2018). By including boys in HPV vaccination programs, boys and men will contribute to herd immunity and thereby help to protect women; the greatest potential health benefits are ultimately for women, because they bear the largest HPV-related disease burden. This difference in potential health gains is significant, because it will partially determine the kinds of vaccination that are applicable. In particular, there are two main areas of tension, which generally revolve around the asymmetry of the HPV disease burden.

First, when it comes to women, the government may take a self-protective approach by making the vaccine available for women and encouraging its uptake, thereby allowing them to protect themselves—yet doing nothing to enforce it.^12^ On the other hand, the government may opt to take the decision out of women’s hands by means of a paternalistic approach—for instance, by mandating HPV vaccination for women. Respect for autonomy favors the former choice, while duties of benevolence (Beauchamp, 2003) and providing equal access to basic healthcare (Verweij and Houweling, 2014) may justify the latter approach.

Second, in the case of men, another tension emerges.^13^ The government may opt for an altruistic approach by encouraging men to vaccinate against HPV for the sake of women, for instance by means of persuasion. It may emphasize duties to contribute to herd immunity (Dawson, 2007; Giubilini *et al.*, 2018) or emphasize concern for sexual partners and the potential benefits that current and/or future partners stand to gain (Luyten *et al.*, 2014). There are also benefits for men that can be stressed in a self-protective approach, but given the substantial asymmetry of disease burden and the relatively small risk of HPV-related cancers for men, such an approach is unlikely to be compelling enough to obtain the necessary vaccination coverage. The altruistic approach has the virtue of respecting autonomy and freedom of choice. Alternatively, the government may take an indirect approach, for instance in the form of mandatory HPV vaccination for boys, thus ultimately taking the decision out of the hands of men. This might be done to minimize the incidence of HPV and thus to reduce the risk of HPV-related cancers especially in women. Since men contribute at least as much to the spread of HPV infections as women, it may be justified to require men to be vaccinated against HPV primarily for the sake of women.^14^

The move from altruistic to indirect vaccination requires a different defense from the altruistic approach, as it considerably constrains individual autonomy. It also requires a stronger ethical justification than the paternalistic approach for women, because it lacks the defense that it is in the best interest of those bearing the burdens of vaccination (that is, boys and men). Which is not to say, of course, that such an intervention is always indefensible.

The case of HPV vaccination reveals the following. First, it is very important to know what the effects of a given vaccine actually are. There is unlikely to be debate about altruistic or indirect approaches to tetanus vaccination, because the effects simply do not extend to people other than those who are vaccinated, thus precluding discussion of other-directed motives. As mentioned earlier, one might vaccinate to be healthy for others (e.g., for one’s children or other dependents). However, keeping oneself healthy in this particular case is part of a more general phenomenon; when specifically considering tetanus vaccination, the primary motive is still likely to be to receive the benefits of vaccination for one’s own person—even if others might also benefit from the things that one is ultimately able to do through good health. Nevertheless, unlikely as it might be, should one’s primary motive for tetanus vaccination truly be to benefit others, then this might yet qualify as altruistic vaccination. From the perspective of vaccination programs, of course, this is still unlikely to a fruitful approach. Vaccines like those against different strands of HPV will take center stage in reflections on altruistic and indirect vaccination, precisely because of the significant effects that those vaccines are likely to have beyond the vaccinated individual.

Second, it is important how governments understand actual and potential vaccination programs. It will help governments to ask what they ultimately want from a vaccination program. Who are supposed to be the primary beneficiaries of the vaccine in question? Who is to receive it? And should people be kept free in their decision-making regarding vaccine uptake, or should some form of pressure or coercion be applied? The answers to these questions, as I have argued, will lead toward one kind of vaccination or the other, along with specific ethical considerations. Even if there are mixed motives, as there may well be, I think that for most vaccines it is—or will at some point be—clear where the largest disease burden lies and who will, accordingly, benefit the most. Examining vaccination from a framework of action–reasons rather than mere actions (Grill, 2007) may also help to clarify the motives and normative issues at stake. In any case, governments should have a clear vision of the motives behind vaccination programs.

Third, it is significant to know the motives of people who are actually or potentially involved in vaccination programs. If, for instance, people are already (potentially) inclined to vaccinate for the sake of others in particular cases (e.g., for current and/or future sexual partners), then it makes sense for governments to explore an altruistic approach—harnessing extant feelings of altruism—before taking an indirect approach, which would limit freedom of decision. If, on the other hand, one finds very little inclination to vaccinate for others, then an altruistic approach may turn out to be ineffective.

Finally, it matters how governments frame the kind of vaccination under consideration to the public. If the (perceived) burdens of vaccination are very high, and the (perceived) benefits to others are very low, then framing vaccination in terms of altruism may gain little traction. Likewise, if the benefits of a particular vaccine to an individual are negligible, then self-protection is clearly not an appropriate way to frame vaccination in this case.

In the end, vaccination policy is unlikely to be static. It will need to be adjusted over time to shifting patterns of disease incidence and vaccine availability, safety and acceptance—acceptance both in the service of one’s own health as well as that of others. What will remain more constant are the larger moral questions, which are intimately related to the particular kinds of vaccination in question.

## Conclusion

I have distinguished different kinds of vaccination along the lines of self- and other-directed motives, and I have argued for the importance of clarifying the structure of the relations that hold between the vaccination decision-maker, the vaccine recipient and the primary beneficiary. Accordingly, I classified four kinds of vaccination: self-protective, paternalistic, altruistic and indirect. Each of these kinds of vaccination evokes a particular set of ethical issues, so that the ethical justification of vaccination is served by clarifying which kind is being considered.

More specifically, moral reflection on vaccination for the sake of others is best approached by first distinguishing between its two forms: the freely opted altruistic kind of vaccination and an imposed kind of indirect vaccination. Governments ought to carefully consider these two kinds of vaccination—and the particular ethical considerations foregrounded by each—when deciding on an approach to take for the regulation of vaccination in the interest of third parties. To this end, it will be fruitful for empirical research to examine what particular target groups (e.g., those who might vaccinate altruistically) consider to be relevant motives, and how much weight they give to these. While my taxonomy provides a solid basis for discussions about self- and other-directed motives, it will benefit from being fleshed out further—both through conceptual refinements as discussions persist, as well as by incorporating relevant empirical data.

## Footnotes

1 Throughout this article, when I refer simply to ‘vaccination’ I mean an instance of individual vaccination, whereas by ‘vaccination program’ I mean a program of collective vaccination.

2 I will not provide additional arguments for this assumption, taking it as uncontroversial that the purpose of preventive vaccination is protection against disease.

3 For the sake of simplicity, I use the singular form for each; as will become clear later, each element may comprise more than a single individual.

4 The classification is not meant to be exhaustive; should further elements be recognized or different kinds be conceived, there is no reason why they could not be integrated.

5 Henceforth I will drop the qualifier ‘probabilistically’, making the point here that vaccination decisions are always based on calculations of probability under conditions of uncertainty.

6 I use the singular for the sake of simplicity; the agent could be a party of agents (e.g., parents/guardians of a child), and the vaccinated person could be more than one (e.g., a group of children).

7 That is, autonomous in their ability to make competent and fully informed (vaccination) decisions. There might be other ways in which children, for instance, are autonomous (e.g., in their movements). Here and elsewhere, when I refer to autonomous agents, I mean agents who are autonomous specifically in their decision-making capacity.

8 Benefits may include, for instance, low doctor's costs associated with having a healthy child, while burdens may include the cost of taking one's child to a pediatrician.

9 These examples are not meant to be complete, nor can they be discussed in the detail they deserve; their purpose is more modest, namely to illustrate altruistic vaccination.

10 It must be noted that in this and in other examples, the vaccinated party often also benefits from the vaccine (Chu and Englund, 2018; Kachikis *et al.*, 2018). What matters for my account is who receives the intended primary benefits.

11 ‘Indirect vaccination’ does not capture the subject of vaccination in the way that self-protective (i.e., the self), paternalistic (i.e., the subject of paternalism) and altruistic (i.e., the subject of altruism) vaccination do. This is in a sense unfortunate; it is also inevitable, and speaks to both the complexity of indirect vaccination and to the importance of further specifying the relevant actors.

12 The case is slightly more complex, because HPV vaccination is often recommended for minors (Kim and Goldie, 2009). Granting adolescent self-consent (Agrawal and Morain, 2018) may avoid issues of autonomy, but even without consent, my approach can inform the discussion through paternalistic vaccination.

13 HPV vaccination is also important for men who have sex with men (Wheldon *et al.*, 2017). I limit my discussion to relations between men and women for reasons of space.

14 Thanks to Marcel Verweij for this point.

## References

[phaa005-B1] Agrawal S., Morain S. R. (2018). Who Calls the Shots? The Ethics of Adolescent Self Consent for HPV Vaccination. Journal of Medical Ethics, 44, 531–535. 29478033 10.1136/medethics-2017-104694

[phaa005-B2] Beauchamp T. L. (2003). Methods and Principles in Biomedical Ethics. Journal of Medical Ethics, 29, 269–274. 14519835 10.1136/jme.29.5.269 PMC1733784

[phaa005-B3] Beauchamp T. L., Childress J. F. (2012). Principles of Biomedical Ethics. 7th edn Oxford: Oxford University Press.

[phaa005-B4] Betsch C. (2014). Overcoming Healthcare Workers’ Vaccine Refusal—Competition between Egoism and Altruism. Eurosurveillance, 19, 1–5. 10.2807/1560-7917.es2014.19.48.20979 25496574

[phaa005-B5] Bray F., Ferlay J., Soerjomataram I., Siegel R. L., Torre L. A., Jemal A. (2018). Global Cancer Statistics 2018: GLOBOCAN Estimates of Incidence and Mortality Worldwide for 36 Cancers in 185 Countries. CA: A Cancer Journal for Clinicians, 68, 394–424. 30207593 10.3322/caac.21492

[phaa005-B6] Broadbent A. (2019). Philosophy of Medicine. Oxford: Oxford University Press.

[phaa005-B7] Carson P. J., Flood A. T. (2017). Catholic Social Teaching and the Duty to Vaccinate. The American Journal of Bioethics, 17, 36–43. 10.1080/15265161.2017.1284914 28328377

[phaa005-B8] Chu H. Y., Englund J. A. (2014). Maternal Immunization. Vaccines, **15**, 560–568. 10.1093/cid/ciu327 PMC4168293 24799324

[phaa005-B9] Conly S. (2013a). Coercive Paternalism in Health Care: Against Freedom of Choice. Public Health Ethics, 6, 241–245.

[phaa005-B10] Conly S. (2013b). Against Autonomy: Justifying Coercive Paternalism. Cambridge: Cambridge University Press. 10.1136/medethics-2013-101444 24335658

[phaa005-B11] Dawson A. (2007). Herd Protection as a Public Good: Vaccination and Our Obligations to Others In Dawson A. and Verweij M. (eds), Ethics, Prevention, and Public Health. Oxford: Oxford University Press, pp. 160–178.

[phaa005-B12] Donken R., King A. J., Bogaards J. A., Woestenberg P. J., Meijer C. J. L. M., de Melker H. E. (2018). High Effectiveness of the Bivalent Human Papillomavirus (HPV) Vaccine against Incident and Persistent HPV Infections up to 6 Years after Vaccination in Young Dutch Women. The Journal of Infectious Diseases, 217, 1579–1589. 29409034 10.1093/infdis/jiy067

[phaa005-B13] Dubov A., Phung C. (2015). Nudges or Mandates? The Ethics of Mandatory Flu Vaccination. Vaccine, 33, 2530–2535. 25869886 10.1016/j.vaccine.2015.03.048

[phaa005-B14] Feemster K. A. (2018). Vaccines: What Everyone Needs to Know. Oxford: Oxford University Press.

[phaa005-B15] Fine P., Eames K., Heymann D. L. (2011). ‘ Herd Immunity’: A Rough Guide. Clinical Infectious Diseases, 52, 911–916. 21427399 10.1093/cid/cir007

[phaa005-B16] Galanakis E., Jansen A., Lopalco P. L., Giesecke J. (2013). Ethics of Mandatory Vaccination for Healthcare Workers. Euro Surveillance, 45, 1–8. 10.2807/1560-7917.es2013.18.45.20627 24229791

[phaa005-B17] Giubilini A. (2019). The Ethics of Vaccination. Cham: Palgrave Macmillan. 30888744

[phaa005-B18] Giubilini A., Douglas T., Savulescu J. (2018). The Moral Obligation to Be Vaccinated: Utilitarianism, Contractualism, and Collective Easy Rescue. Medicine, Health Care, and Philosophy, 21, 547–560. 10.1007/s11019-018-9829-y PMC6267229 29429063

[phaa005-B19] Grill K. (2007). The Normative Core of Paternalism. Res Publica, 13, 441–458.

[phaa005-B20] Healy C. M., Rench M. A., Baker C. J. (2011). Implementation of Cocooning against Pertussis in a High-Risk Population. Clinical Infectious Diseases, 52, 157–162. 21288837 10.1093/cid/ciq001

[phaa005-B21] Houweling H., Verweij M., Ruitenberg E. J. (2010). Criteria for Inclusion of Vaccinations in Public Programs. Vaccine, 28, 2924–2931. 20189486 10.1016/j.vaccine.2010.02.021

[phaa005-B22] Kachikis A., Eckert L. I., Englund J. (2018). Who’s the Target: Mother or Baby? Viral Immunology, 31, 184–194. 29474132 10.1089/vim.2017.0135

[phaa005-B23] Kim J. J., Goldie S. J. (2009). Cost Effectiveness Analysis of Including Boys in a Human Papillomavirus Vaccination Programme in the United States. BMJ, 339, 1–10. 10.1136/bmj.b3884 PMC2759438 19815582

[phaa005-B24] Krantz I., Sachs L., Nilstun T. (2004). Ethics and Vaccination. Scandinavian Journal of Public Health, 32, 172–178. 15204177 10.1080/14034940310018192

[phaa005-B25] Kraut R. (2009). What Is Good and Why: The Ethics of Well-Being. Cambridge: Harvard University Press.

[phaa005-B26] Kraut R. (2016). In Zalta E. N. (ed), *“Altruism.” the Stanford Encyclopedia of Philosophy*, available from: https://plato.stanford.edu/entries/altruism/ [accessed 17 November 2019].

[phaa005-B27] Luyten J., Engelen B., Beutels P. (2014). The Sexual Ethics of HPV Vaccination for Boys. HEC Forum, 26, 27–42. 23907594 10.1007/s10730-013-9219-z

[phaa005-B28] Metcalf C. J. E., Ferrari M., Graham A. L., Grenfell B. T. (2015). Understanding Herd Immunity. Trends in Immunology, 36, 753–755. 26683689 10.1016/j.it.2015.10.004

[phaa005-B29] Munoz F. M. (2018). Current Challenges and Achievements in Maternal Immunization Research. Frontiers in Immunology, 9, 1–7. 29559976 10.3389/fimmu.2018.00436 PMC5845678

[phaa005-B30] Navin M. (2016). Values and Vaccine Refusal. New York: Routledge.

[phaa005-B31] Omer S. B., Salmon D. A., Orenstein W. A., deHart P., Halsey N. (2009). Vaccine Refusal, Mandatory Immunization, and the Risks of Vaccine-Preventable Diseases. The New England Journal of Medicine, 360, 1981–1988. 19420367 10.1056/NEJMsa0806477

[phaa005-B68751497] Orenstein W. A., Ahmed R. (2017). Simply Put: Vaccination Saves Lives. Proceedings of the National Academy of Sciences of the United States of America, **114**, 4031–4033. 28396427 10.1073/pnas.1704507114 PMC5402432

[phaa005-B32] Pierik R. (2018). Mandatory Vaccination: An Unqualified Defence. Journal of Applied Philosophy, 35, 381–398.

[phaa005-B33] Plans-Rubió P. (2012). The Vaccination Coverage Required to Establish Herd Immunity against Influenza Viruses. Preventive Medicine, 55, 72–77. 22414740 10.1016/j.ypmed.2012.02.015

[phaa005-B34] Schiller J. T., Castellsague X., Garland S. M. (2012). A Review of Clinical Trials of Human Papillomavirus Prophylactic Vaccines. Vaccine, 30, F123–F138. 23199956 10.1016/j.vaccine.2012.04.108 PMC4636904

[phaa005-B35] Schwartz J. L., Caplan A. L. (2011). Ethics of Vaccination Programs. Current Opinion in Virology, 1, 263–267. 22440783 10.1016/j.coviro.2011.05.009

[phaa005-B36] Scott N., Seglow J. (2007) Altruism. Berkshire: Open University Press.

[phaa005-B37] Seglow J. (2004). Altruism and Freedom In Seglow, J. (ed.) The Ethics of Altruism. London: Frank Cass Publishers, pp. 145–171.

[phaa005-B38] Sipp D., Frazer I. H., Rasko J. E. J. (2018). No Vacillation on HPV Vaccination. Cell, 172, 1163–1167. 29522737 10.1016/j.cell.2018.02.045

[phaa005-B39] Urwyler P., Heininger U. (2014). Protecting Newborns from Pertussis—The Challenge of Complete Cocooning. BMC Infectious Diseases, 14, 12. 25037057 10.1186/1471-2334-14-397 PMC4223593

[phaa005-B40] Van Delden J. J. M., Ashcroft R., Dawson A., Marckmann G., Upshur R., Verweij M. F. (2008). The Ethics of Mandatory Vaccination against Influenza for Health Care Workers. Vaccine, 26, 5562–5566. 18722495 10.1016/j.vaccine.2008.08.002

[phaa005-B41] Van Panhuis W. G., Grefenstette J., Jung S. Y., Chok N. S., Cross A., Eng H., Lee B. Y., Zadorozhny V., Brown S., Cummings D., Burke D. S. (2013). Contagious Diseases in the United States from 1888 to the Present. The New England Journal of Medicine, 369, 2152–2158. 24283231 10.1056/NEJMms1215400 PMC4175560

[phaa005-B42] Van Wijhe M., McDonald S. A., de Melker H. E., Postma M. J., Wallinga J. (2016). Effect of Vaccination Programmes on Mortality Burden among Children and Young Adults in the Netherlands during the 20th Century: A Historical Analysis. The Lancet Infectious Diseases, 16, 592–598. 26873665 10.1016/S1473-3099(16)00027-X

[phaa005-B43] Verweij M. (2005). Obligatory Precautions against Infection. Bioethics, 19, 323–335. 16222840 10.1111/j.1467-8519.2005.00446.x

[phaa005-B44] Verweij M., Houweling H. (2014). What Is the Responsibility of National Government with Respect to Vaccination? Vaccine, 32, 7163–7166. 25454880 10.1016/j.vaccine.2014.10.008

[phaa005-B45] Verweij M., Lambach P., Ortiz J. R., Reis A. (2016). Maternal Immunization: Ethical Issues. The Lancet Infectious Diseases, 16, e310–e314. 27663129 10.1016/S1473-3099(16)30349-8 PMC8243647

[phaa005-B46] Wheldon C. W., Daley E. M., Buhi E. R., Baldwin J. A., Nyitray A. G., Giuliano A. G. (2017). HPV Vaccine Decision-Making among Young Men Who Have Sex with Men. Health Education Journal, 76, 52–65. 10.1177/0017896916647988 PMC11451994 39371403

[phaa005-B47] World Health Organization (2013). Global Vaccine Action Plan: 2011–2020. Geneva: WHO Press, available from: http://www.who.int/immunization/global_vaccine_action_plan/GVAP_doc_2011_2020/en/ [accessed 2 February 2020].

